# Development and psychometric testing of a theory-based tool to measure self-care in diabetes patients: the Self-Care of Diabetes Inventory

**DOI:** 10.1186/s12902-017-0218-y

**Published:** 2017-10-16

**Authors:** Davide Ausili, Claudio Barbaranelli, Emanuela Rossi, Paola Rebora, Diletta Fabrizi, Chiara Coghi, Michela Luciani, Ercole Vellone, Stefania Di Mauro, Barbara Riegel

**Affiliations:** 1 Department of Medicine and Surgery, University of Milan-Bicocca, Monza, Italy; 2 Department of Psychology, University La Sapienza, Rome, Italy; 3 Centre of Biostatistics for Clinical Epidemiology, Department of Medicine and Surgery, University of Milan-Bicocca, Monza, Italy; 4 ASST Niguarda, Milan, Italy; 5 ASST Vimercate, Vimercate, Italy; 6 Department of Biomedicine and Prevention, University of Rome Tor Vergata, Rome, Italy; 7 University of Pennsylvania, Philadelphia, PA USA; 8 Via Cadore 48, 20900 Monza, Italy

**Keywords:** Self-care, Self-efficacy, Diabetes mellitus, Psychometric testing, Middle range theory, Chronic disease

## Abstract

**Background:**

Self-care is essential for patients with diabetes mellitus. Both clinicians and researchers must be able to assess the quality of that self-care. Available tools have various limitations and none are theoretically based. The aims of this study were to develop and to test the psychometric properties of a new instrument based on the middle range-theory of self-care of chronic illness: the Self-Care of Diabetes Inventory (SCODI).

**Methods:**

Forty SCODI items (5 point Likert type scale) were developed based on clinical recommendations and grouped into 4 dimensions: self-care maintenance, self-care monitoring, self-care management and self-care confidence based on the theory. Content validity was assessed by a multidisciplinary panel of experts. A multi-centre cross-sectional study was conducted in a consecutive sample of 200 type 1 and type 2 diabetes patients. Dimensionality was evaluated by exploratory factor analyses. Multidimensional model based reliability was estimated for each scale. Multiple regression models estimating associations between SCODI scores and glycated haemoglobin (HbA1c), body mass index, and diabetes complications, were used for construct validity.

**Results:**

Content validity ratio was 100%. A multidimensional structure emerged for the 4 scales. Multidimensional model-based reliabilities were between 0.81 (maintenance) and 0.89 (confidence). Significant associations were found between self-care maintenance and HbA1c (*p* = 0.02) and between self-care monitoring and diabetes complications (*p* = 0.04). Self-care management was associated with BMI (*p* = 0.004) and diabetes complications (*p* = 0.03). Self-care confidence was a significant predictor of self-care maintenance, monitoring and management (all *p* < 0.0001).

**Conclusion:**

The SCODI is a valid and reliable theoretically-grounded tool to measure self-care in type 1 and type 2 DM patients.

**Electronic supplementary material:**

The online version of this article (doi: 10.1186/s12902-017-0218-y) contains supplementary material, which is available to authorized users.

## Background

The prevalence of diabetes mellitus is increasing worldwide. It is estimated that 8.2% of adults aged 20 to 79 years have diabetes, for a total of 387 million people globally [1]. This number is predicted to rise to more than 592 million in 2035 [2]. Diabetes and its complications are a principal cause of morbidity (e.g. cardiovascular disease, renal disease, retinopathy, and neuropathy) and premature death [3]. Promoting self-care can improve this dismal picture in those with type 1 (T1DM) or type 2 (T2DM) diabetes [4, 5].

Self-care of diabetes includes eating in a healthy manner, being physically active, monitoring blood glucose, taking medications, solving problems as they occur, reducing risks, and coping in a healthy fashion [6]. Blood pressure monitoring, weight monitoring, and activities intended to manage the symptoms of hyper- and hypoglycaemia are also needed [4, 6]. Adequate self-care improves metabolic control [7] and quality of life [8], and reduces cardiovascular risk [9], hospitalizations [10] and disease related complications [11].

The availability of valid and reliable tools to assess diabetes self-care is fundamental to identifying patients at risk of poor outcomes [5]. At least 16 tools assessing diabetes self-care or self-care related constructs are available [12]. Ten are unidimensional, focusing exclusively on diet, physical activity, blood glucose monitoring, oral care or insulin management [13]. Another six multidimensional tools are available to measure diabetes self-care [14–19]. The two most commonly used with adults are the Summary of Diabetes Self-Care Activities (SDSCA) and the Self-Care Inventory Revised (SCI-R) [15, 16]. Both instruments were developed before 2005 and have not been updated with recent clinical information [12, 13]. Only 2 of the 6 instruments used an explicit theoretical framework and they are not intended to measure diabetes self-care behaviours (diabetes-related distress and diabetes self-efficacy) [20, 21]. Although all 6 instruments used exploratory factor analysis, internal consistency reliability did not account for dimensionality of the tools [12]. Criterion validity was mostly estimated using scores from other scales rather than clinical indicators [12, 13]. These limitations have been noted in recent systematic reviews noting that none of the instruments available to measure diabetes self-care have evidence of a strong validation process or a theoretical grounding [12, 13]. This picture illustrates the need for a theoretically grounded, psychometrically-sound instrument measuring diabetes self-care.

The purpose of this study was to develop and test the psychometric properties of a new instrument based on the middle range-theory of self-care of chronic illness [22]: the *Self-Care of Diabetes Inventory* (SCODI). According to that theory, self-care is defined as a process of maintaining health through health promoting practices and managing illness. Self-care is performed in both healthy and ill states. The key concepts addressed in the theory include self-care maintenance, self-care monitoring, and self-care management. Confidence in the ability to perform self-care is important in each stage of the self-care process [22–24]. Consistent with methodological recommendations, specific objectives of this study were to evaluate SCODI: 1) content validity; 2) dimensionality and internal coherence; and 3) construct validity [25].

## Methods

First, SCODI items were developed using the theoretical framework. Then, content validity of items was evaluated by a multidisciplinary panel of experts. Next, a multicentre cross-sectional study was conducted to test the psychometric properties of the SCODI. The Institutional Review Boards of ASST Grande Ospedale Metropolitano Niguarda and Policlinico di Monza approved the study. General, Health and Nursing Directors of the two centres authorized patient recruitment and data collection. All participants provided written informed consent. Participants were asked to think about their self-care during the prior month. Details about both development and testing are described below.

### Theoretical framework

Self-care maintenance reflects those behaviours used to preserve health, maintain physical and emotional stability, or improve well-being. Self-care maintenance includes illness-related behaviours, such as adherence to follow-up visits and examinations, and health promoting behaviours, such as eating a healthy diet or engaging in physical activity. Healthy behaviours performed to avoid disease and complications are included in self-care maintenance as well [22].

Self-care monitoring is a process of routine, vigilant body monitoring, surveillance, or “body listening” [22]. Self-care monitoring includes symptom recognition and interpretation. The goal of self-care monitoring is recognition that a change has occurred (e.g. symptoms) and the correct interpretation about when to take action. Patients who are skilled in self-care monitoring know when to communicate with a healthcare professional to obtain timely and appropriate care. Self-care monitoring is the link between self-care maintenance and self-care management [22].

Self-care management is a process of responding with appropriate behaviours to health changes and problems to avoid an exacerbation. Self-care management entails treatment implementation and evaluation of treatment. Based on the required skills, the response can be simple or complex. Self-care management behaviours may be implemented directly by the patient (autonomous) or in consultation with a health care provider (consultative) [22].

Self-care confidence is not an element of self-care but a factor that strongly influences self-care maintenance, self-care monitoring, and self-care management [22]. Self-care confidence reflects the degree of confidence that a patient has about his or her ability to perform a specific self-care-related task. As such, self-care confidence reflects self-efficacy or the ability to perform a specific action and persist in performing that action or behaviour, despite barriers or challenges [22].

### Scale development and content validity

A literature review was performed to identify best practice recommendations about self-care behaviours of T1DM and T2DM diabetes patients. Recent international clinical guidelines were reviewed [4, 5] and the main evidence-based recommendations were used to develop the 40 SCODI items. According to the theoretical framework, SCODI items were grouped to reflect self-care maintenance (12 items), self-care monitoring (8 items), self-care management (9 items) and self-care confidence (11 items). A multidisciplinary panel of experts (*n* = 15) with diabetologists (*n* = 5), diabetes nurses (*n* = 6), a psychologist (*n* = 1) and academic researchers (*n* = 3) evaluated content validity.

Content validity is the extent to which the domain of interest is comprehensively sampled by items in the questionnaire [25]. The content of a questionnaire is valid when a clear description is provided for the measurement aim, the target population and the concepts being measured, and when experts were involved in item selection [25]. For content validity of the SCODI, the theoretical framework was used to clarify the measurement aim and concepts. Experts evaluated the relevance of each SCODI item using a 4-point Likert scale. Content validity index was calculated for each item as the proportion of experts who rated it as relevant or very relevant (3 or 4). Content validity for the 4 scales was calculated as the proportion of items rated as relevant or very relevant (3 or 4) within the total items of each separate scale. Minor changes and revisions suggested by the panel of experts were incorporated. Content validity index of the final version of each item and each scale was 100%. The full final SCODI English Version is available as Additional file 1 **.**

### Sample and setting

A consecutive sample of adults with diabetes was recruited from diabetes outpatient services of ASST Grande Ospedale Metropolitano Niguarda and Policlinico di Monza in two provinces of northern Italy. Inclusion criteria were: confirmed diagnosis of T1DM or T2DM diabetes diagnosed according to guideline criteria [4]; age ≥ 18 years. Exclusion criteria were: screening or first visit to the diabetes centre; time from the diagnosis of diabetes < 1 year; illiteracy; documented cognitive impairment. A sample of 7 patients per item was needed to allow adequate inference in exploratory or confirmative factor analysis [25]. As noted above, the SCODI was not intended to provide an overall measure of self-care; it was designed as an inventory with 4 separate scales measuring 4 different constructs. Self-care maintenance was the longest scale with 12 items. Thus, a sample of 84 patients would have been adequate to address the main study objective (dimensionality and internal consistency); however, we enrolled 200 participants to support a more stable analysis [25, 26].

### Data collection

Participants completed the *Self-Care of Diabetes Inventory* by self-report during an outpatient visit. Socio-demographic data including age, gender, education, marital status, family income, employment status and caregiver support were collected using a survey developed by the researchers. Clinical data such as medications taken, body mass index (BMI), glycated haemoglobin (HbA1c), presence of diabetes micro-vascular complications (diabetic foot, diabetic kidney disease, diabetic retinopathy, diabetic neuropathy), and presence of co-morbidities, were collected from the medical record by nurse research assistants. Complications were judged by established criteria [4]. To assure the quality of data, data collectors were trained carefully and random data monitoring was performed to verify collected data. Data monitoring involved comparing case report forms used for the study with the patients’ original medical records.

### Data analysis

Sociodemographic and clinical characteristics were described using means, frequencies and percentages. As dimensionality testing must precede reliability testing [27], we began the analysis with factor analysis and then assessed reliability. Factor analyses were conducted using Mplus 7.4 [28] within the Exploratory Structural Equation Modeling approach (ESEM) [29–31]. Compared to confirmatory factor analysis (CFA), ESEM does not require that all or most cross-loadings in the factorial pattern be fixed at zero. This requirement is often too stringent, causing loss of fit and extensive re-specifications with modification indices. Like exploratory factor analysis (EFA), ESEM does not require that the factor pattern be specified in advance, since all indicators depend on all factors [31]. However, as opposed to EFA, ESEM provides all the usual SEM parameters (e.g. residual variances and co-variances) and factor loadings, standard errors, and tests of goodness of fit. Since the SCODI has never undergone factor analysis before and the number of participants in the analysis was limited, we examined dimensionality using the more exploratory approach of ESEM.

The SCODI uses a 5 point Likert type scale where higher scores indicate better self-care. Since the SCODI item response format uses only 5 ordered categories and the data are not normally distributed, we used a Weighted Least Square method, WLS-MV estimator [28], which is recommended for ordinal or dichotomous variables [32]. Using a multifaceted approach to assessment of model fit [33], we considered the following goodness of fit indices. Comparative Fit Index (CFI) .90–.95 indicates acceptable fit, > .95 indicates good fit. Root Mean Square Error of Approximation (RMSEA) ≤ .05 indicates a well-fitting model, .05–.08 indicates moderate fit, ≥.10 indicates poor fit. RMSEA with 90% confidence intervals (≤ .05 to ≤.08) indicates good fit. The test of close-fit examines the probability that the approximation error is low (*p*-values > .05 indicate good fit). Standardized Root Mean Square Residual (SRMR) ≤ .08 indicates good fit [34]. Additionally, traditional chi-square statistics are reported. However, due to the sample size and the sensitivity of the chi-square likelihood ratio test to sample size, chi-square test results were not used in interpreting model fit.

Consistent with recent developments [27], reliability was computed with the composite reliability or omega coefficient [35]. Alpha assumes that the items reflect a unidimensional structure. Knowing that there are several dimensions represented in a scale, a more appropriate reliability coefficient that takes into account the multidimensionality of the scale is the *global reliability index for multidimensional scales* [36]. Although the dimensionality of a scale is complex, as noted by Bentler [37] “every multidimensional coefficient implies a particular composite with maximal unidimensional reliability” (p. 343). Thus, the final reliability estimates derived with appropriate methods “can be interpreted to represent a unidimensional composite” [37] (p. 341).

For aim 3 we estimated construct validity following Terwee’s recommendations [25]. We tested these theoretical hypotheses: 1) if SCODI measures the concepts described in the theoretical framework, self-care confidence will be significantly associated with self-care maintenance, self-care monitoring, and self-care management; 2) if SCODI measures the self-care behaviors of diabetic patients, significant associations will exist between self-care maintenance, self-care monitoring, and self-care management scores and one or more clinical indicators theoretically influenced by patients’ self-care behaviors. A set of external clinical indicators was identified from the literature describing clinical outcomes associated with diabetes self-care [4]: BMI, HbA1c, and microvascular diabetes complications (diabetic foot, diabetic kidney disease, diabetic retinopathy, diabetic neuropathy).

To test the first hypothesis, the Pearson correlation coefficient was estimated and three quantile regression models were run to explain the effect of self-care confidence, adjusting for type of diabetes, age, gender, education, family income, caregiver support and time from diagnosis of diabetes on self-care maintenance, self-care monitoring and self-care management.

To test the second hypothesis, linear (BMI and HbA1c) and logistic (presence of microvascular diabetes complications) regression models were run on self-care maintenance, self-care monitoring and self-care management scores, adjusting for gender, age at enrolment, type of diabetes, time from the diagnosis of diabetes (<10 and ≥10 years), and presence of comorbidities.

## Results

Of the 200 enrolled participants, 75% had T2DM (*n* = 150) and 25% had T1DM (*n* = 50). Most were female, retired, and reporting a low annual income (Table 1).

**Table 1:** Sociodemographic and clinical characteristics of the sample (*n* = 200)

	T1DM patients (*n* = 50) n (%)	T2DM patients (*n* = 150) n (%)	Total *n* = 200 n (%)
Gender			
Male	30 (60.0)	53 (35.3)	83 (41.5)
Female	20 (40.0)	97 (64.7)	117 (58.5)
Age			
20–39 years	28 (56.0)	3 (2.0)	31 (15.5)
40–59 years	20 (40.0)	34 (22.7)	54 (27.0)
60–79 years	2 (4.0)	92 (61.3)	94 (47.0)
80–99 years	0 (0.0)	21 (14.0)	21 (10.5)
Educational level			
Elementary-Middle School	8 (16)	107 (71.3)	115 (57.5)
High School-University Degree	42 (84)	43 (28.7)	85 (42.5)
Occupation			
Working	46 (92.0)	37 (24.7)	83 (41.5)
Retired	3 (6.0)	107 (71.3)	110 (55.0)
Unemployed	1 (2.0)	6 (4.0)	7 (3.5)
Exemption for low income			
Yes	10 (20.0)	85 (56.7)	95 (47.5)
No	40 (80.0)	65 (43.3)	105 (52.5)
Time from the diagnosis of diabetes			
1–9 years	10 (20.0)	73 (48.7)	83 (41.5)
10–19 years	14 (28.0)	47 (31.3)	61 (30.5)
20–49 years	26 (52.0)	30 (20.0)	56 (28.0)
Body Mass Index			
Normal weight	29 (58.0)	27 (18.0)	56 (28.0)
Overweight	17 (34.0)	55 (36.7)	72 (36.0)
Obesity	4 (8.0)	68 (45.3)	72 (36.0)
Number of medications			
0	1 (2.0)	2 (1.3)	3 (1.5)
1–3	39 (78.0)	29 (19.3)	68 (34.0)
≥ 4	10 (20.0)	119 (79.3)	129 (64.5)
HbA1c			
5.0%(31 mmol/mol) - 6.9%(52 mmol/mol)	16 (32.0)	59 (39.3)	75 (37.5)
7.0% (53 mmol/mol)- 7.9%(63 mmol/mol)	14 (28.0)	54 (36.0)	68 (34.0)
8.0%(64 mmol/mol) - 15.9%(150 mmol/mol)	20 (40.0)	37 (24.7)	57 (28.5)
Presence of at least one diabetes microvascular complications			
Yes	18 (36.0)	60 (40.0)	78 (39.0)
No	32 (64.0)	90 (60.0)	122 (61.0)
Diabetes retinopathy			
Yes	16 (32.0)	19 (12.7)	35 (17.5)
No	34 (68.0)	131 (87.3)	165 (82.5)
Diabetes kidney Disease			
Yes	3 (6.0)	23 (15.3)	26 (13.0)
No	47 (94.0)	127 (84.7)	174 (87.0)
Diabetic foot			
Yes	2 (4.0)	5 (3.3)	7 (3.5)
No	48 (96.0)	145 (96.7)	193 (96.5)
Diabetes neuropathy			
Yes	3 (6.0)	28 (18.7)	31 (15.5)
No	47 (94.0)	122 (81.3)	169 (84.5)
Presence of at least one co-morbidity			
Yes	26 (52.0)	144 (96.0)	170 (85.0)
No	24 (48.0)	6 (4.0)	30 (15.0)

Note. HbA1C between 5.0% and 6.9% is controlled; between 7.0% and 7.9% is uncontrolled; between 8.0% and 15.9% is severally uncontrolled

### Self-care maintenance

#### Dimensionality

A series of ESEM was conducted on the 12 items of the self-care maintenance scale, testing a 1 to a 4 factor model. Of these models, only the 4 factor model had adequate fit indices (Table 2 **)**. Factor loadings of the 4 factor solution, taken from the Mplus output for completely standardized solutions, are reported in Table 3. Factor loadings were generally medium to high, attesting to a substantial proportion of common variance among items. Factor correlations were low (Factor 1/Factor 2 = −0.01; Factor 1/Factor 3 = −0.08; Factor 1/Factor 4 = −0.35; Factor 2/Factor 3 = 0.16; Factor 2/Factor 4 = 0.26; Factor 3/Factor 4 = 0.24), indicating a moderate association among the different facets of self-care maintenance. Factors within self-care maintenance were labeled as: health promoting exercise behaviors (Factor 1), disease prevention behaviors (Factor 2); health promoting behaviors (Factor 3), and illness-related behaviors (Factor 4). Factors labeling is discussed below.

**Table 2:** Fit indices for the 4 SCODI scales derived from ESEM

	χ2	DF	p(χ2)	TLI	CFI	SRMR	RMSEA. .10 Confidence Internal, p(RMSEA < .05)
Self-care maintenance scale (4 factors)	27.52	24	.28	.98	.99	.047	.059, [.0, .066], *p* = .17
Self-care monitoring scale (2 factors)	56.33	13	.001	.98	.99	.072	.129 [.10, .17], *p* < .001
Self-care management scale (2 factors)	15.53	13	.28	.99	.99	.040	.031, [.0, .08], *p* = .68
Self-care confidence scale (2 factors)	62.24	34	.01	.97	.98	.059	.064, [.038, .089], *p* = .17

Note. *DF* degree of freedom, *TLI* Tucker Lewis Index, *CFI* comparative fit index, *RMSEA* Root Mean Square Error of Approximation

**Table 3:** Exploratory factor analysis and item factor loadings for the self-care maintenance, self-care monitoring, self-care management and self-care confidence scales

Self-care maintenance	Factor 1 loadings	Factor 2 loadings	Factor 3 loadings	Factor 4 Loadings
1. Maintain an active life-style (example: walking, going out, doing activities)?	**0.60**	0.06	0.36	−0.03
2. Perform physical exercise for 2 h and 30 min each week (example: swimming, going to the gym, cycling, walking)?	**0.98**	0.01	0.00	0.30
3. Eat a balanced diet of carbohydrates (pasta, rice, sugars, bread), proteins (meat, fish, legumes), fruits and vegetables?	0.11	−0.12	**0.50**	0.31
4. Avoid eating salt and fats (example: cheese, cured meats, sweets, red meat)?	0.03	−0.09	**0.35**	0.12
5. Limit alcohol intake (no more than 1 glass of wine/day for women and 2 glasses/day for men)?	0.04	**0.78**	0.01	0.06
6. Try to avoid getting sick (example: wash your hands, get recommended vaccinations)?	−0.24	**0.39**	0.35	0.03
7. Avoid cigarettes and tobacco smoke?	0.01	**0.72**	−0.08	−0.07
8. Take care of your feet (wash and dry the skin, apply moisture, use correct socks)?	−0.11	−0.01	**0.78**	−0.10
9. Maintain good oral hygiene (brush your teeth at least twice/day, use mouthwash, use dental floss)?	0.14	0.29	**0.31**	−0.05
10. Keep appointments with your health care provider?	−0.13	0.02	−0.06	**0.89**
11. Have your health check-ups on time? (example: blood tests, urine tests, ultrasounds, eye exams)?	−0.01	0.02	0.08	**0.93**
12. Many people have problems taking all their prescribed medicines. Do you take all your medicines as your health care provider prescribed (please also consider insulin if your doctor prescribed it for you)?	−0.44	0.25	**0.37**	0.15
Self-care monitoring	Factor 1 loadings	Factor 2 loadings		
13. Monitor your blood sugar regularly?	**0.56**	0.26		
14. Monitor your weight?	**0.54**	−0.02		
15. Monitor your blood pressure?	**0.69**	−0.19		
16. Keep a record of your blood sugars in a diary or notebook?	**0.41**	0.21		
17. Monitor the condition of your feet daily to see if there are wounds, redness or blisters?	**0.42**	0.11		
18. Pay attention to symptoms of high blood sugar (thirst, frequent urination) and low blood sugar (weakness, perspiration, anxiety)?	0.36	**0.66**		
19. How quickly did you recognize that you were having symptoms?	−0.007	**0.94**		
20. How quickly did you know that your symptoms were due to diabetes?	0.000	**0.99**		
Self-care management	Factor 1 loadings	Factor 2 loadings		
21. Check your blood sugar when you feel symptoms (such as thirst, frequent urination, weakness, perspiration, anxiety)?	**0.89**	0.002		
22. When you have abnormal blood sugar levels, do you take notes about the events that could have caused it and actions you took?	−0.07	**0.74**		
23. When you have abnormal blood sugar levels, do you ask a family member or friend for advice?	−0.09	**0.44**		
24. When you have symptoms, and you discover that your blood sugar is low, do you eat or drink something with sugar to solve the problem?	**0.68**	−0.01		
25. If you find out that your blood sugar is high, do you adjust your diet to fix it?	0.28	**0.46**		
26. If you find out that your blood sugar is high, do you adjust your physical activity to fix it?	0.06	**0.59**		
27. After taking actions to adjust an abnormal blood sugar level, do you re-check your blood sugar to assess if the actions you took were effective?	**0.68**	0.32		
28. If you find out that your blood sugar is very low or very high, do you call your health care provider for advice?	0.01	**0.62**		
Self-care confidence	Factor 1 loadings	Factor 2 loadings		
30. Prevent high or low blood sugar levels and its symptoms.	**0.55**	0.14		
31.Follow advice about nutrition and physical activity.	0.31	**0.36**		
32. Take your medicines in the appropriate way (including insulin if prescribed).	0.15	**0.41**		
33. Persist in following the treatment plan even when it’s difficult.	−0.004	**0.75**		
34. Monitor your blood sugar as often as your health care provider asked you to.	**0.55**	0.07		
35. Understand if your blood sugar levels are good or not.	**0.84**	−0.19		
36. Recognize the symptoms of low blood sugar.	**0.79**	−0.35		
37. Persist in monitoring your diabetes even when it’s difficult.	0.002	**0.83**		
38. Take action to adjust your blood sugar and relieve your symptoms.	**0.80**	0.08		
39. Evaluate if your actions were effective to change your blood sugar and relieve your symptoms.	**0.88**	0.005		
40. Persist in carrying out actions to improve your blood sugar even when it’s difficult.	0.36	**0.63**		

Note. Item 29 asking “If you find out that your blood sugar is too high or too low, do you adjust your insulin dosage in the way your health care provider suggested?” was excluded by this analysis to maintain an adequate sample size because only patients taking insulin answer the question. However, it was included in the scoring of the scale when applicable to estimate internal consistency and construct validity. Thus, we recommend including this item when scoring Factor 2 labelled as “Consultative self-care management behaviours” and especially when scoring the whole Self-care management scale in people taking insulin. Bold is used to indicate where each SCODI item showed the more significant factor loading (>.3 or higher)

#### Internal coherence

The internal consistency reliability of the four factors representing self-care maintenance were all high attesting to the internal coherence of those dimensions (Factor 1 = 0.85; Factor 2 = 0.77; Factor 3 = 0.79; Factor 4 = 0.95). However, the self-care maintenance scale was intended to yield a single score, not four different scores. When the alpha coefficient was computed for the full scale, a poor coefficient of .55 was obtained. When the *global reliability index for multidimensional scales* was used, reliability was .81 for the overall self-care maintenance scale (Table 4).

**Table 4:** Internal consistency reliability of single factors and overall SCODI scales

	Single factor reliability	Multidimensional model based reliability
Self-care maintenance		0.81
Health promoting exercise behaviours	0.85	
Disease prevention behaviours	0.79	
Health promoting behaviours	0.77	
Illness related behaviours	0.95	
Self-care monitoring		0.84
Body listening	0.72	
Symptom recognition	0.99	
Self-care management		0.86
Autonomous self-care management behaviours	0.89	
Consultative self-care management behaviours	0.77	
Self-care confidence		0.89
Task-specific self-care confidence	0.90	
Persistence self-care confidence	0.88	

### Self-care monitoring

#### Dimensionality

A series of ESEM were conducted on the 8 items of the self-care monitoring scale, testing respectively a 1 to 3 factor models. The best fitting model was a 3 factor solution; however, this solution presented a residual factor with only one item with a clear primary loading. Thus, the less fitting but more interpretable 2-factor solution was selected. This solution had adequate fit indices (Table 2). Factor loadings of the 2-factor solution were generally high attesting to a substantial proportion of common variance among the items (Table 3 **)**. The correlation between the two factors was non-significant (Factor 1/Factor 2 = 0.06). Factors were labeled as body listening (Factor 1) and symptom recognition (Factor 2).

#### Internal coherence

The internal consistency reliability of the two factors representing self-care monitoring were high, attesting to the internal coherence of the two dimensions (Factor 1 = 0.72; Factor 2 = 0.99). The self-care monitoring scale was intended to yield a single score, but the alpha coefficient was .65. When the *global reliability index for multidimensional scales* was used, reliability was .84 for the overall self-care monitoring scale (Table 4).

### Self-care management

#### Dimensionality

When ESEM was conducted on the 8 items of the self-care management scale, the best fitting model was the 2-factor solution (Table 2). The factor loadings of the 2-factor solution were high (Table 3 **).** The two factors were significantly correlated at .55. Factors were labeled as autonomous self-care management behaviors (Factor 1) and consultative self-care management behaviors (Factor 2).

#### Internal coherence

Internal consistency reliability of the two self-care management factors was high (Factor 1 = 0.89; Factor 2 = 0.77). When alpha coefficient was computed for the full scale, an adequate coefficient of .77 was obtained. However, reliability was .86 for the overall self-care management scale using the *global reliability index for multidimensional scales* (Table 4).

### Self-care confidence

#### Dimensionality

A series of ESEM conducted on the 11 items of the self-care confidence scale yielded a 3-factor solution; however, one factor had only one item with a clear primary loading. Thus, the less fitting but more interpretable 2-factor solution was selected. This solution had generally excellent fit indices (Table 2 **)**. Factor loadings of the 2-factor solution were high (Table 3 **)**. The correlation of the two factors was .55. Factors were labeled as task specific self-care confidence (Factor 1) and persistence self-care confidence (Factor 2).

#### Internal coherence

The internal consistency reliabilities of the two self-care confidence factors were all high (Factor 1 = 0.90; Factor 2 = 0.88). When alpha coefficient was computed for the full 11item scale an adequate coefficient of .80 was obtained. When the *global reliability index for multidimensional scales* was computed, the reliabiilty was .89 for the overall self-care confidence scale (Table 4).

### Construct validity

The correlation coefficients of self-care confidence versus self-care maintenance, self-care monitoring and self-care management were 0.4, 0.51 and 0.53 respectively. Self-care confidence was also significantly associated with all three self-care scale scores in the multivariable model (self-care maintenance beta = 0.30 *p* < .0001, self-care monitoring beta = 0.71 *p* < .0001, and self-care management beta = 0.79 *p* < .0001). Higher levels of self-care maintenance were significantly associated with lower HbA1c (beta = −0.02, *p* = 0.025), higher self-care monitoring with the presence of diabetes complications (Odds Ratio = 1.02, *p* = 0.043) and higher self-care management with both lower BMI (beta = −0.06, *p* = 0.004) and the absence of diabetes complications (Odds Ratio = 0.98, *p* = 0.03).

## Discussion

The purpose of this study was to develop and to test the psychometric properties of a new instrument measuring the self-care of persons with diabetes mellitus. Using the middle range theory of chronic illness, we developed an instrument to measure self-care maintenance, monitoring, and management, and demonstrated content validity, reliability and construct validity. An additional scale measuring self-care confidence, a factor shown to predict successful self-care in other populations and to be important in predicting outcomes, is also available. Although this study was conducted in Italy, the sociodemographic and clinical profile of the enrolled patients aligns with the international literature [38], suggesting that the SCODI may be useful in other contexts. Overall, the results of this study suggest that SCODI is a valid and reliable instrument that can be useful to others wishing to measure self-care in persons with diabetes.

The SCODI addresses the limitations of other available instruments. To our knowledge, this is the first theory-based instrument measuring self-care behaviors of diabetic patients—the most important limitation of previous tools [12, 13]. Use of the most recent clinical recommendations in the development process and the high content validity index suggest that the SCODI is up-to-date clinically, addressing a second major problem of other tools [12, 13]. Construct validity was estimated using strong clinical indicators, addressing another limitation—the failure to validate against clinical criteria. The results of this study provide support for the middle range theory of self-care of chronic illness [22]. To our knowledge, this is the first time that the middle-range theory of chronic illness has been used in diabetes.

The factorial structure and the low inter-factor correlations of the self-care maintenance scale are not surprising because theoretically, self-care maintenance is known to be a complex and multifaceted dimension of self-care [22, 23]. The self-care maintenance factors reflect the theoretical framework, as we found health promoting self-care maintenance behaviours, disease prevention self-care maintenance behaviors, and illness-related behaviors as described in the theory [22]. Health promoting behaviors addressed: diet (item 3 and 4), foot care (item 8), oral hygiene (item 9) and adherence to prescribed medications (item 12). In essence, this factor includes adherence to all the main diabetes treatments.

In a diabetes population, it is not surprising that exercise behaviors (Factor 1, including being active and exercise) aggregated into a unique factor, even though these are “health promoting behaviors” as well. Previous studies show that physical activity in diabetes patients is particularly poor and dependent on complex psychological and social factors [39, 40]. That is why we found two dimensions of self-care maintenance health promoting behaviors: one addressing physical activity and one addressing all the other recommended behaviors. Logically, it is easy to anticipate that patients may attend to diet and medicine-taking without engaging in physical activity, even if both are necessary elements of self-care. The factors that motivate exercise probably differ from those that motivate the other health promotion behaviors.

Disease prevention behaviors related to alcohol consumption, smoking habits, hand hygiene and vaccinations clustered as a separate factor in the self-care maintenance scale. This factor matches with both the theory and diabetes self-management education standards, where all these behaviors are described as fundamental to avoid complications, disease exacerbation, and severe co-morbidities [5]. Items addressing adherence to follow-up visits and check-up examinations grouped into the illness-related self-care maintenance factor. These behaviors are needed only if requested by a healthcare provider [4].

The factorial structure and the low inter-factor correlation of the self-care monitoring scale represented the two main areas of self-care monitoring: body listening and symptom recognition. This dimension had adequate but unsatisfying fit indices. The 2-factor solution represented the main elements of self-care monitoring as described theoretically [22], so a 2-factor solution was chosen. The body listening factor included items about: glucose monitoring and record keeping, weight monitoring, blood pressure monitoring, and foot monitoring. The second factor included items asking about the attention paid to symptoms and the time needed to recognize them.

The self-care management scale split into autonomous and consultative behaviors that patients with diabetes need to do when responding to changes and problems [22]. Behaviors such as checking blood sugar if a symptom is recognized (item 21), eating sugar when blood sugar is low (item 24), and re-checking blood sugar after a remedy is tried (item 27) represents the basic recommendations given to patients with diabetes [5]. These items grouped together and were labeled autonomous self-care management behaviors. Other behaviors such as taking notes about the events that can alter blood glucose levels (item 22), asking for advice from formal or informal caregivers (item 23 and 28), modifying diet, exercise and/or insulin to adjust blood sugar levels represent complex, reflective and skilled activities of self-care management often performed in consultation with a provider. These items as a group were named consultative self-care management behaviors.

Self-care confidence is the degree of confidence that a person has about his or her ability to perform a specific self-care task and to persist in performing an action despite barriers, consistent with accepted definitions of self-efficacy [22]. The factorial structure of the self-care confidence scale displayed two factors representing confidence about specific tasks (item 30, 34, 35, 36, 38, 39) and persistence (item 31, 32, 33, 37, 40). Based on this, we labeled these two factors as task specific self-care confidence and persistence self-care confidence.

The theoretical hypotheses tested to assess construct validity were both supported. Patients with higher self-care confidence performed better self-care maintenance, self-care monitoring and self-care management and good correlations were found between self-care confidence and the other three scales. These results confirms the theoretical relationships expressed in the theoretical framework [22]. Furthermore, the same associations were found in previous studies using a diabetes self-efficacy scale to estimate the impact of self-efficacy on behaviors and outcomes of diabetes patients [41]. Higher self-care maintenance was associated with better HbA1c levels. Previous studies found significant associations between adherence to medications, diet or exercise and glycemic control in diabetes patients [7]. All these behaviors are addressed in the self-care maintenance scale and clinically they are known to improve glycemic control in diabetes patients [4]. Higher self-care monitoring was associated with more diabetes complications. People feeling severely ill or having a more compromised health status are more likely to perform better self-care [42]. Previous studies found similar results studying blood glucose monitoring in diabetes [38]. Finally, higher self-care management was associated with fewer diabetes complications and lower BMI [4]. The association of self-care with objective clinical indicators is one of the most important criteria for the external validity of a health instrument [25], thus these results provide strong evidence of the validity of the SCODI. As we adjusted for the main confounding variables, including the type of diabetes, these associations also support the usefulness of the SCODI measures in both type 1 and type 2 diabetes patients.

Overall, the high single factor and global model-based internal reliabilities of the 4 self-care scales allow researchers and clinicians to use overall scales scores (e.g. self-care maintenance) or to calculate a specific score for each of the subscales (e.g., health promoting behaviors). In fact, the single factors reliability of each subscale (i.e. health promoting exercise behaviors in the self-care maintenance scale) were all good enough (all ≥ 0.77) to allow single factor scoring. Thus, single factor scores can be used to allow researchers and clinicians to look at specific aspects (i.e. body listening, symptom recognition) and to tailor individualized self-care promotion interventions. Scores for each scale and subscale should be standardized to a 0–100 scale to facilitate comparisons. We strongly discourage users from calculating a total SCODI score. Instead, the data will be far more useful if scores are calculated for each individual scale (self-care maintenance, self-care monitoring, self-care management and self-care confidence).

This study has several limitations. The sample was enrolled in one country and the sample size, although adequate for the study objectives, was relatively limited. Thus, usefulness of the SCODI in other settings cannot be assumed. However, high content validity was estimated by a panel of experts, multiple centers were represented and the patient characteristics are aligned with the global diabetes population, suggesting that the SCODI may be useful elsewhere. SCODI testing is ongoing in Europe, in China and both in South and North America. The inclusion of both T1DM and T2DM patients represents both a strength and a limitation of this study. On one side, this is coherent with the study population (patients with DM) and type of diabetes was used to adjust all the construct validity associations. This supports our conclusion that the SCODI is valid and reliable in the population of patients with DM. On the other side, psychometric performances could show specificities in the two subgroups (T1DM and T2DM). Thus, future research should include confirmatory factor analysis focusing on T1DM and T2DM samples.

## Conclusion

Self-care is essential for patients with diabetes mellitus. Both clinicians and researchers need to be able to assess the quality of that self-care. The SCODI has been shown to be a valid and reliable tool to measure self-care in patients with diabetes mellitus and could be useful to both clinicians and researchers. Clinicians can use a theory-based approach to better understand patients’ self-care and to tailor specific interventions aimed to improve one or more of the self-care processes. Furthermore, clinicians could use the theory-based language to measure, document, and communicate where the patient is having a specific problem. For researchers, the three processes of self-care (maintenance, monitoring, and management) and self-care confidence have never been described in the diabetes population. Further research is needed to describe them, to identify their determinants, to study outcomes associated with the processes, and to develop specific tailored healthcare interventions. Furthermore, as the SCODI was developed based on a theory of chronic illness already in use to study other diseases, the SCODI could contribute to a more general understanding of living with chronic conditions. Empirical data from the SCODI could corroborate and further develop the middle-range theory.

## Additional file

Additional file 1: The Self-Care of Diabetes Inventory (SCODI) English Version.This Additional file reports the full Self-Care of Diabetes Inventory (SCODI) English Version, where Section A represents the self-care maintenance scale; Section B represents the self-care monitoring scale; Section C represents the self-care management scale; and, Section D represents the self-care confidence scale. (PDF 562 kb)

## Abbreviations

BMI: Body mass index
CFA: Confirmatory factor analysis
DM: Diabetes mellitus
EFA: Exploratory factor analysis
ESEM: Exploratory structural equation modelling
HbA1c: Glycated haemoglobin
SCODI: Self-Care of Diabetes Inventory
T1DM: Type 1 diabetes mellitus
T2DM: Type 2 diabetes mellitus
