# Supplementary material for: Development and psychometric testing of a theory-based tool to measure self-care in diabetes patients: the Self-Care of Diabetes Inventory
Source: BMC Endocr Disord. 2017 Oct 16;17:66. doi: 10.1186/s12902-017-0218-y (PMC5644085; doi:10.1186/s12902-017-0218-y)
Supplement: Additional file 1: — The Self-Care of Diabetes Inventory (SCODI) English Version.This Additional file reports the full Self-Care of Diabetes Inventory (SCODI) English Version, where Section A represents the self-care maintenance scale; Section B represents the self-care monitoring scale; Section C represents the self-care management scale; and, Section D represents the self-care confidence scale. (PDF 562 kb) [file 12902_2017_218_MOESM1_ESM.pdf]

Self-Care of Diabetes Inventory (SCODI)

Please think about what you did and how you felt in the last month.

SECTION A

Below are listed some behaviors that a person with diabetes could perform to maintain health and wellness. Please indicate how often or routinely you do these behaviors.

(circle **one** number)

		NEVER				ALWAYS
1.	Maintain an active life-style (example: walking, going out, doing activities)?	1	2	3	4	5
2.	Perform physical exercise for 2 hours and 30 minutes each week? (example: swimming, going to the gym, cycling, walking)	1	2	3	4	5
3.	Eat a balanced diet of carbohydrates (pasta, rice, sugars, bread), proteins (meat, fish, legumes), fruits and vegetables?	1	2	3	4	5
4.	Avoid eating salt and fats (example: cheese, cured meats, sweets, red meat)?	1	2	3	4	5
5.	Limit alcohol intake (no more than 1 glass of wine/day for women and 2 glasses/day for men)?	1	2	3	4	5
6.	Try to avoid getting sick (example: wash your hands, get recommended vaccinations)?	1	2	3	4	5
7.	Avoid cigarettes and tobacco smoke?	1	2	3	4	5
8.	Take care of your feet (wash and dry the skin, apply moisture, use correct socks)?	1	2	3	4	5
9.	Maintain good oral hygiene (brush your teeth at least twice/day, use mouthwash, use dental floss)?	1	2	3	4	5
10.	Keep appointments with your health care provider?	1	2	3	4	5
11.	Have your health check-ups on time? (example: blood tests, urine tests, ultrasounds, eye exams)?	1	2	3	4	5
12.	Many people have problems taking all their prescribed medicines. Do you take all your medicines as your health care provider prescribed (please also consider insulin if your doctor prescribed it for you)?	1	2	3	4	5

SECTION B

Below are some behaviors that a person with diabetes could practice to monitor their diabetes. Please indicate how often you do these behaviors.

(circle **one** number)

		NEVER				ALWAYS
13.	Monitor your blood sugar regularly?	1	2	3	4	5
14.	Monitor your weight?	1	2	3	4	5
15.	Monitor your blood pressure?	1	2	3	4	5
16.	Keep a record of your blood sugars in a diary or notebook?	1	2	3	4	5
17.	Monitor the condition of your feet daily to see if there are wounds, redness or blisters?	1	2	3	4	5
18.	Pay attention to symptoms of <u>high</u> blood sugar (thirst, frequent urination) and <u>low</u> blood sugar (weakness, perspiration, anxiety)?	1	2	3	4	5

Note. If you used in the last month a **continuous blood glucose monitor**, please answer **5** at item #13 and #16.

The last time you had symptoms:

		I DIDN'T RECOGNIZE SYMPTOMS	NOT QUICKLY				VERY QUICKLY
19.	How quickly did you recognize that you were having symptoms?	0	1	2	3	4	5
20.	How quickly did you know that your symptoms were due to diabetes?	0	1	2	3	4	5

SECTION C

Below are listed some behaviors a person with diabetes could do to improve their blood sugar when it's too high or too low. How often you do (or you would do) these actions when symptoms occur or when your blood sugar is out of range?

(circle **one** number)

		NEVER				ALWAYS
21.	Check your blood sugar when you feel symptoms (such as thirst, frequent urination, weakness, perspiration, anxiety)?	1	2	3	4	5
22.	When you have abnormal blood sugar levels, do you take notes about the events that could have caused it and actions you took?	1	2	3	4	5
23.	When you have abnormal blood sugar levels, do you ask a family member or friend for advice?	1	2	3	4	5
24.	When you have symptoms, and you discover that your blood sugar is <u>low</u>, do you eat or drink something with sugar to solve the problem?	1	2	3	4	5
25.	If you find out that your blood sugar is <u>high</u>, do you adjust your diet to fix it?	1	2	3	4	5
26.	If you find out that your blood sugar is <u>high</u>, do you adjust your physical activity to fix it?	1	2	3	4	5
27.	After taking actions to adjust an abnormal blood sugar level, do you re-check your blood sugar to assess if the actions you took were effective?	1	2	3	4	5
28.	If you find out that your blood sugar is very <u>low</u> or very <u>high</u>, do you call your health care provider for advice?	1	2	3	4	5

Do you take insulin?

- ☐ Yes
- ☐ No

If yes, please answer the following question.

29.	If you find out that your blood sugar is too high or too low, do you adjust your insulin dosage in the way your health care provider suggested?	1	2	3	4	5

SECTION D

People with diabetes have to develop skills to take care of themselves and to maintain their health. How confident do you feel doing the following activities?

(circle **one** number)

		NOT CONFIDENT AT ALL				EXTREMELY CONFIDENT
30.	Prevent high or low blood sugar levels and its symptoms.	1	2	3	4	5
31.	Follow advice about nutrition and physical activity.	1	2	3	4	5
32..	Take your medicines in the appropriate way (including <u>insulin</u> if prescribed).	1	2	3	4	5
33.	Persist in following the treatment plan even when it's difficult.	1	2	3	4	5
34.	Monitor your blood sugar as often as your health care provider asked you to.	1	2	3	4	5
35.	Understand if your blood sugar levels are good or not.	1	2	3	4	5
36.	Recognize the symptoms of low blood sugar.	1	2	3	4	5
37.	Persist in monitoring your diabetes even when it's difficult.	1	2	3	4	5
38.	Take action to adjust your blood sugar and relieve your symptoms.	1	2	3	4	5
39.	Evaluate if your actions were effective to change your blood sugar and relieve your symptoms.	1	2	3	4	5
40.	Persist in carrying out actions to improve your blood sugar even when it's difficult.	1	2	3	4	5

Thank you for completing this survey!
